# Screening potential reference genes for quantitative real-time PCR analysis in the oriental armyworm, *Mythimna separata*

**DOI:** 10.1371/journal.pone.0195096

**Published:** 2018-04-04

**Authors:** Hong-Bo Li, Chang-Geng Dai, Chang-Rong Zhang, Yong-Fu He, Hai-Yan Ran, Shi-Hong Chen

**Affiliations:** Institute of Plant Protection, Guizhou Academy of Agricultural Science, Guiyang, China; Chinese Academy of Agricultural Sciences Institute of Plant Protection, CHINA

## Abstract

The oriental armyworm, *Mythimna separata*, is a major insect pest in China and other Asian countries. Unfortunately, suitable reference genes for quantitative real-time PCR (qRT-PCR) have not been previously identified in *M*. *separata* for normalizing target gene expression. In this study, we evaluated the expression stability of eight candidate genes (*18S*, *ACT*, *EF1-α*, *GAPDH*, *RPS7*, *RPS13*, *RPL32* and *TUB*) in *M*. *separata* using the comparative ΔCt method, BestKeeper, Normfinder geNorm and ReFinder, a comprehensive software platform. The results indicated that the appropriate reference gene varied depending on the experimental conditions. We found that *ACTIN*, *EF1-α* and *TUB* were optimal for different developmental stages; *TUB*, *RPS13* and *EF1-α* showed the most stable expresssion in different tissues; *RPS13* and *18S* were the best reference genes for monitoring expression under high temperature conditions; *TUB*, *RPS13* and *RPS7* exhibited the most stable expression under larval-crowding conditions; *RPS7*, *EF1-α*, *RPL32* and *GAPDH* were the best for pesticide exposure experiments. This study provides tools for reliable normalization of qRT-PCR data and forms a foundation for functional studies of target gene expression in *M*. *separata*.

## Introduction

Choosing the appropriate reference gene(s) is critical for assessing the accuracy of target gene expression using quantitative real-time PCR (qRT-PCR) analysis [1]. Ideally, a reference gene should be expressed constantly in samples subjected to selected experimental conditions [2,3]. Recent studies have shown that reference genes have variable expression levels under different biotic (e.g. diverse insect tissues and developmental stages) and abiotic conditions (e.g. temperatures, pesticides, different photoperiods) [4–9]. Yang et al. identified V-ATPase A as the most stable reference gene for different developmental stages in *Coleomegilla maculata*; however, this gene was the least stable among different *C*. *maculata* sexes and dsRNA treatments [10]. Pan et al. identified *GAPDH* as the most suitable reference gene for tissues and insect sex in *Hippodamia convergens*, but it was least suitable for temperature stress [11]. Numerous studies now suggest that there is no single ‘universal’ reference gene that can be used for diverse experimental conditions, even within a single species [12–15]. Therefore, it is essential to evaluate the expression stability of reference genes under different experimental treatments before normalizing target gene expression.

The oriental armyworm, *Mythimna separata* (Walker) (Lepidoptera: Noctuidae) is a migratory insect pest in China and other Asian countries. Currently, *M*. *separata* is a serious threat for corn production in China [16–18]. Due to its economic importance, the migratory behavior and regulation of *M*. *separata* has been documented [19, 20]. More recently, the development of next-generation sequencing technologies and transcriptome analysis has provided a huge amount of genetic information for expression studies. For example, Liu et al. used transcriptome analysis to identify a large number of genes involved in pesticide resistance, insect development and the stress response in *M*. *separata* [21]. Li et al. identified many differentially expressed genes involved in circadian rhythm, hormone regulation, energy metabolism and neurotransmitter receptor pathways by comparing the transcriptomes of migrant and resident *M*. *separata* [22]. Furthermore, Chang et al. [23] and Liu et al. [24] identified numerous genes with putative roles in olfactory, sensory and taste transduction by transcriptional analysis of gene expression in the heads and antennae of *M*. *separata*. Quantitative examination of gene expression can further our understanding of the molecular mechanisms underlying insect development, the stress response, and behavioral regulation in *M*. *separata* and may identify novel targets for controlling this pest.

Actin has been used as a reference gene to normalize the expression of target genes involved in the development, stress response and behavior of *M*. *separata* [21, 25–26]. However, these studies did not evaluate the suitability of this gene under different experimental treatments, which may lead to incorrect conclusions regarding the biological functions of these target genes in *M*. *separata*. In this study, we utilized the comparative ΔCt method [14], BestKeeper [27, 28], NormFinder [29], and geNorm [13] and a comprehensive software platform-RefFinder [30] to evaluate the stability of eight commonly-used reference genes (18S ribosomal RNA (*18S*), ACTIN (*ACT*), elongation factor 1 alpha (*EF-1α*), glyceraldehyde-3-phosphate dehydrogenase (*GAPDH)*, ribosomal protein S7 (*RPS7*), ribosomal protein S13 *(RPS13*), ribosomal protein L32 (*RPL32*) and tubulin (*TUB*)) during *M*. *separata* development, in different *M*. *separata* tissues and in response to abiotic (high temperatures and pesticide exposure) and biotic stress (larval crowding). Heat-shock protein 90 (Hsp90) is an abundant and highly conserved molecular chaperone that is induced by multiple environmental factors. We then evaluated the different reference genes using *M*. *separata* heat shock protein 90 (*Mshsp90*) as a target gene to validate our findings. Our results provide a more precise approach to normalize qRT-PCR data in *M*. *separata*, which will improve our understanding of target gene functions in this important pest.

## Materials and methods

### Ethics statement

The *M*. *separata* larvae were collected from corn stalks cultivated in Qianxi county, Guizhou province (27°01′39.72″N, 106°20′2.92″E), in 2015. In present study, there were no specific permits being required for the insect collection. No endangered or protected species were involved in the field studies. The ‘‘List of Protected Animals in China” does not contain the *M*. *separata* which are common insect.

### Insects

The collected larvae (n ≥ 100) were maintained in a climate-controlled chamber at 25 ± 1°C with a 14 L: 10 D photoperiod and 70–80% relative humidity. The larvae were fed with fresh corn leaves until satiated, and then transferred into a plastic box containing wet paper for pupation. Forty newly-emerged pairs were transferred to nylons cage for mating, provided with rice stems for oviposition, and supplied with a 10% (w/v) honey solution.

### Sample treatment and collection

#### Developmental stages and tissues

Developmental stages of *M*. *separata* included 100 eggs, 20 first instar larvae, 10 second instar larvae, and three individuals of the remaining stages (3^rd^-6^th^ instar larvae, pupae and adults). All samples were collected in a microcentrifuge tube (1.5 mL), immediately frozen in liquid nitrogen, and stored at -80°C. Each treatment contained three replications.

Insect tissues (head, epidermis, foregut, midgut, hindgut, and Malpighian tubes) were collected from 6^th^ instar larvae. Tissue collected from three individuals were mixed and constituted one replication, and each treatment was replicated three times.

#### Stress conditions

*M*. *separata* 3^rd^ instar larvae were exposed to elevated temperatures ranging from 31–39°C at 2° increments. Larvae exposed to 24°C constituted the control group. Each temperature consisted of three replications.

Larval crowding trials included five levels: 1, 5, 10, 20, and 30 larvae per plastic bottle (12 x 9 cm, height x diameter). The hatched 1^st^ instar under each densiy were raised as above conditions. When larvae reached the second day of 5^th^ instar (about 15 days), three larvae from same density were collected and considered to be one replication. Each density had three replications.

For insecticide exposure experiments, fresh corn leaves were dipped into five concentrations (0, 1, 2, 4 and 8 μg mL^-1^) of chlorpyrifos for 10 s. Treated leaves were air-dried and placed in glass petri dishes (9 cm diameter). Twelve 3^rd^-instar larvae were transferred into the petri dishes and allowed to feed on treated leaves for 24 h. Survival rates were approximately 100, 95, 70, 40, and 15% for 0, 1, 2, 4 and 8 μg mL^-1^, respectively. Surviving larvae were collected for RNA extraction, and each concentration was replicated three times.

### Quantitative real-time PCR analysis

Total RNA was extracted using the SV Total RNA isolation system (Promega, WI, USA) introduction and treated with Dnase I to eliminate DNA contamination. The integrity and purity of RNA in all samples were verified by agarose gel electrophoresis and spectrophotometric measurements, respectively. Total RNA was reverse-transcribed to cDNA in 20 μL reaction volumes using the iScript gDNA Clear cDNA Synthesis Kit (Bio-Rad, CA, USA), and the cDNA was used as a template for qRT-PCR analysis. PCR was performed in 20 μL reaction volumes containing 10 μL SsoAdvanced Universal SYBR Green Supermix (Bio-Rad), 1 μL of each gene specific primer (Table 1), 1 μL of cDNA template, and 7 μL of ddH_2_O. Reactions were conducted in a CFX96 real-time PCR system (Bio-Rad). The PCR parameters were as follows: 95°C for 3 min, 35 cycles of 95°C for 5 s, and 30 s at the T_m_ of each primer pair (Table 1), followed by melting curve analysis to investigate the specificity of PCR products. Every treatment included three replicates, and each reaction was run in triplicate.

**Table 1:** Primer sequences used in qRT-PCR.

Gene	Accession No.	Primer Sequence (5’-3’)	Size (bp)	T_m_ (°C)	Efficiency (%)	R^2^
*RPS7*	AB669190	F: CGCCAACAAACAGAAGAGGC	219	55.5	93.2	0.997
		R: CGCCCCGTAAGCTTCTTGTA				
*RPS13*	GQ222274	F: ACTGACTGCTGATGATGTGAAGG	77	57.5	106.3	0.996
		R: TGACACCGATTTGGGAGGG				
*18S*	FJ875999.	F: CGGCGACGCATCTTTCAA	85	56.8	105.7	0.997
		R: TTCCCCGTTACCCGTGACA				
*GAPDH*	HM055756	F: AAAATCTCCGTCCTCTCCGA	140	55.8	109.9	0.990
		R: ACCTTCTTGGCACCACCCT				
*ACT*	GQ856238	F: AACTTCCCGACGGTCAAGTCAT	168	60	104.6	0.996
		R: TGTTGGCGTACAAGTCCTTACG				
*TUB*	EU100016	F: CGGTAATGCCTGCTGGGAA	118	54.3	94.0	0.998
		R: CTCGCTGAAGAAGGTGTTGAA				
*RPL32*	AB669190	F: GTGAAAAAGCGGACGAAAAGA	187	53	95.2	0.987
		R: GGAAACCATTGGGCAGCATA				
*EF-1α*	KR869785	F: CTCCACTGAGCCCCCATACA	128	59.4	108.4	0.994
		R: CTCCGTGCCAGCCAGAAAT				

### Statistical analysis

The raw Ct values were obtained using CFX Manager 3.1 (Bio-RAD). The comparative ΔCt method [14], BestKeeper [27–28], NormFinder [29], and geNorm [13] were used to evaluate the stability of the eight candidate reference genes in all treatments. RefFinder (http://150.216.56.64/referencegene.php) was used to estimate and screen the most suitable reference genes by combining the results of the four methods [30]. To obtain the optimal number of reference genes for normalization, geNorm utilizes the V-value (0.15) to decide whether additional reference genes are necessary. If the V-value exceeds 0.15, three reference genes are required for normalization [13]. The analysis procedures followed the instructions provided with the algorithms. For transcriptional analysis, fold-changes in mRNA expression profiles of *Mshsp90* were evaluated using the 2^-ΔΔct^ method [31]. One-way ANOVA was used to detect significances in *Mshsp90* expression levels between treatments, followed by a Duncan's multiple range with *P* ≤ 0.05. All procedures were performed using Data Processing System (DPS) software [32].

## Results

### Primer specificity, efficiency and Ct values

**Fig 1:**
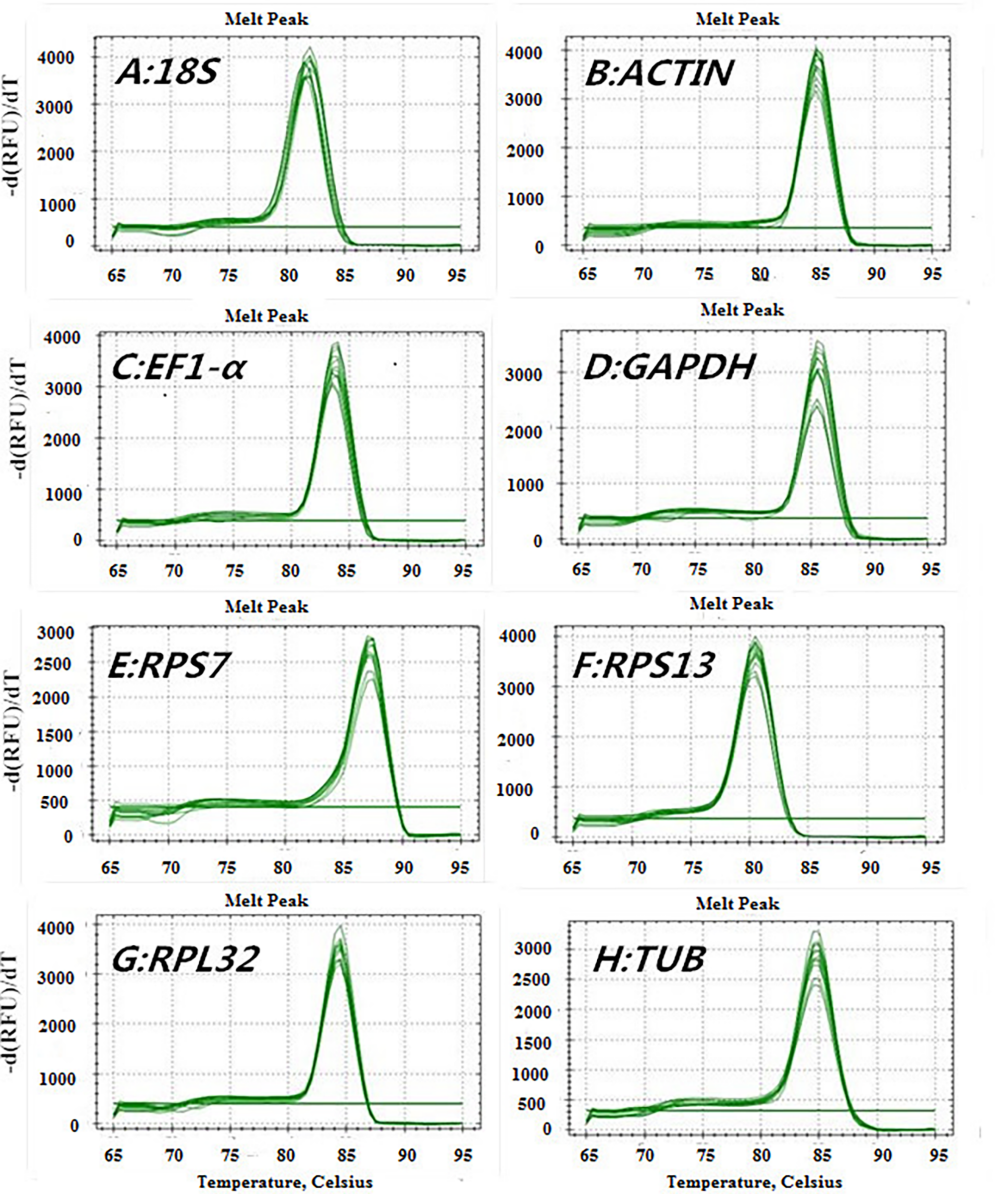
Melting curve analysis of eight candidate reference genes. Genes included: (A) *18S*, (B) *ACT*, (C) *EF1-α*, (D) *GAPDH*, (E) *RPS7*, (F) *RPS13*, (G) *RPL32* and (H) *TUB*.

The eight candidate reference genes were amplified as single bands of the predicted size in 1.5% agarose gels (data not shown). Gene-specific amplification was confirmed by a single peak in melting-curve analysis (Fig 1). Furthermore, the amplification efficiency of all eight genes ranged from 93.2% to 109.9% with correlation coefficients (R^2^) of 98.7% and above (Table 1).

**Fig 2:**
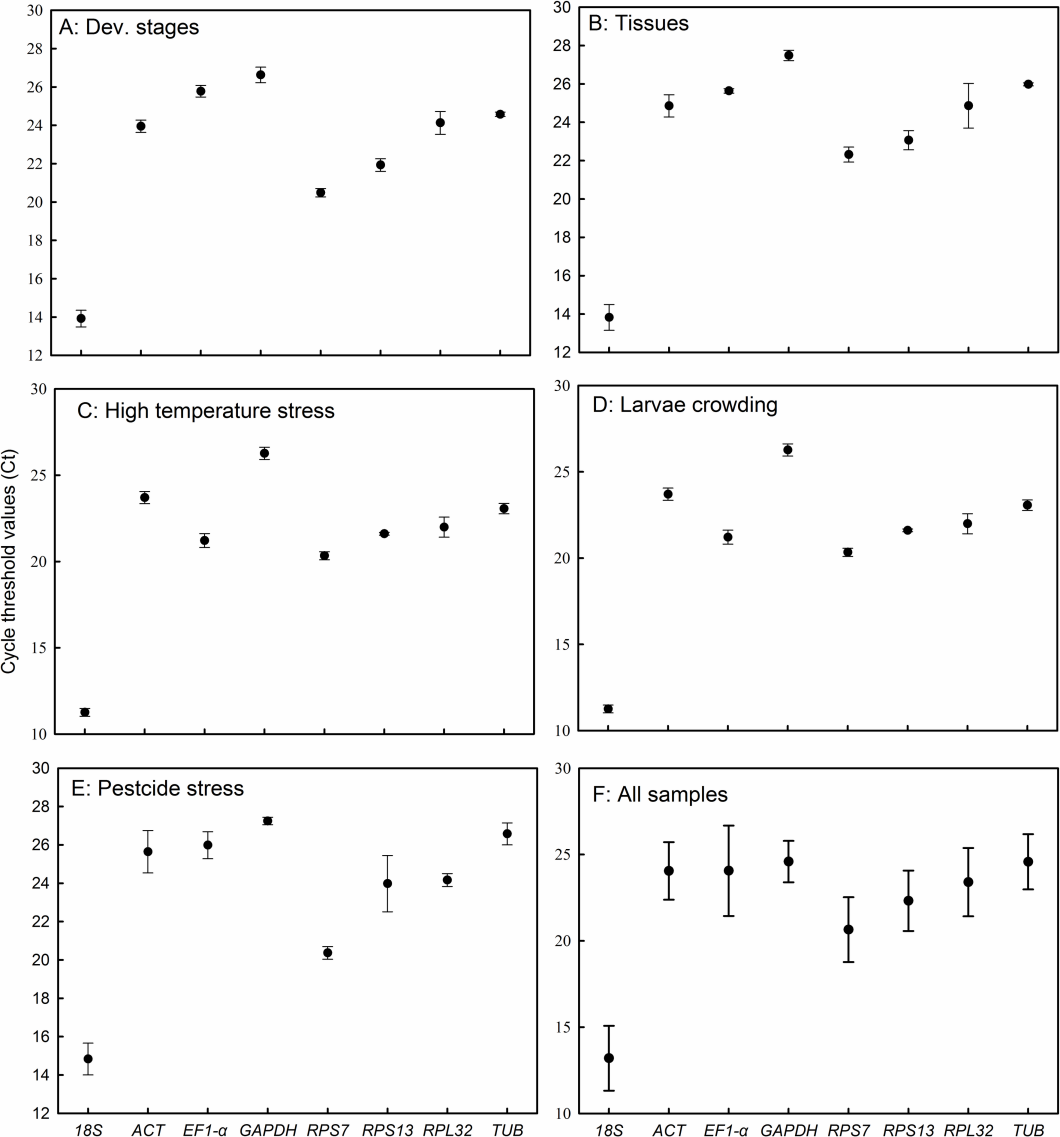
The cycle threshold (Ct) values of eight candidate reference genes. The filled circles indicate the mean Ct values for a given reference gene, and vertical bars represent the standard deviation (SD) of the mean.

To analyze the expression profiles of the eight candidate reference genes, Ct values were calculated for each treatment and all samples, respectively. As shown in Fig 2, Ct values of eight candidate reference genes exhibited relatively different variation for each treatment. Totally, the mean Ct values of the eight genes was less than 25; the lowest and highest mean Ct values were 13.20 for *18S* and 24.59 for *GAPDH*, respectively (Fig 2F).

### Expression in different developmental stages and tissues

**Fig 3:**
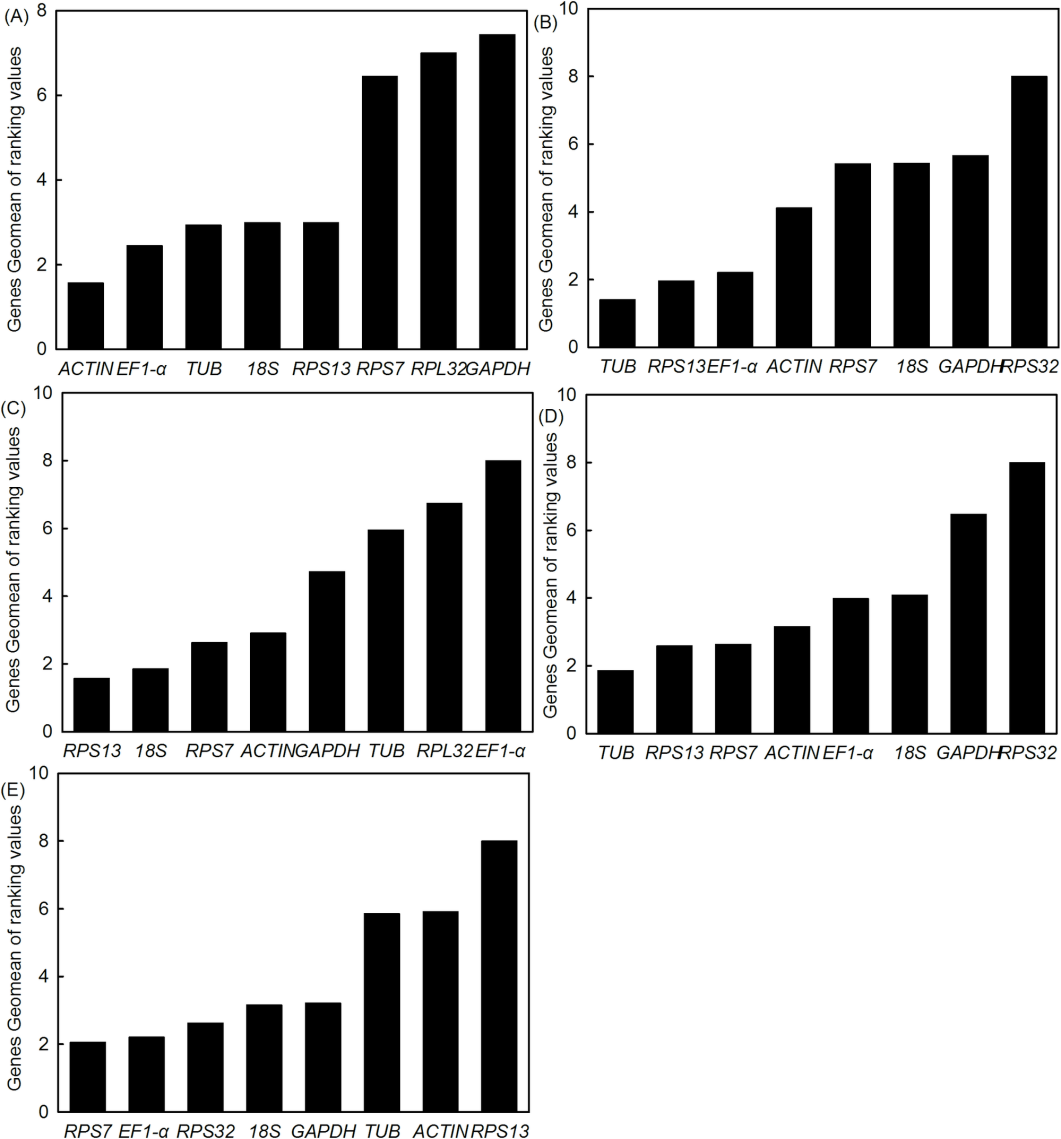
Stability ranking of candidate reference genes as determined by RefFinder. Lower Geomean values indicate more stable expression using RefFinder. Panels represent (A) developmental stages, (B) tissues, (C) high temperature stress, (D) larval crowding, and (E) pesticide exposure.

The stability ranking of the eight different reference genes varied widely among the four methods. The ΔCt and geNorm tools identified *ACT* as the most stable gene, but BestKeeper and NormFinder identified *TUB* and *18S* as most stable, respectively (Table 2). Three methods (ΔCt, NormFinder and geNorm) identified *GAPDH* as the least stable reference gene, while BestKeeper identified *RPS7* as the least stable. When analyzed by RefFinder, the stability order of the reference genes among the different developmental stages was *ACT*>*EF1-α*>*TUB*>*18S*>*RPS13*>*RPS7*>*RPL32*>*GAPDH* (Fig 3A).

**Table 2:** Stability of reference genes in different developmental stages and insect tissues.

Condition	Rank	Δ Ct		BestKeeper		NormFinder		geNorm	
		Gene	stability value	Gene	stability value	Gene	Stability value	Gene	Stability value
Dev. stage	1.00	*ACT*	1.29	*TUB*	0.43	*18S*	0.46	*ACT*	0.53
	2.00	*RPS13*	1.30	*ACT*	0.73	*RPS13*	0.46	*EF-1α*	0.53
	3.00	*EF1-α*	1.31	*RPS13*	0.82	*ACT*	0.48	*TUB*	0.75
	4.00	*18S*	1.31	*GAPDH*	0.83	*EF1-α*	0.53	*RPS13*	0.98
	5.00	*TUB*	1.42	*18S*	0.84	*TUB*	0.67	*18S*	1.07
	6.00	*RPS7*	1.73	*EF-1α*	1.10	*RPL32*	1.11	*RPS7*	1.28
	7.00	*RPL32*	1.80	*RPL32*	1.38	*GAPDH*	1.19	*RPL32*	1.38
	8.00	*GAPDH*	1.91	*RPS7*	1.67	*GAPDH*	1.18	*GAPDH*	1.51
Tissues	1.00	*TUB*	1.21	*TUB*	0.17	*RPS13*	0.25	*TUB*	0.22
	2.00	*RPS13*	1.21	*EF1-α*	0.22	*TUB*	0.58	*EF1-α*	0.22
	3.00	*EF1-α*	1.26	*GAPDH*	0.54	*ACT*	0.66	*RPS13*	0.81
	4.00	*ACT*	1.31	*RPS7*	0.75	*EF1-α*	0.72	*ACT*	0.90
	5.00	*18S*	1.45	*RPS13*	0.80	*18S*	0.97	*18S*	0.97
	6.00	*RPS7*	1.63	*ACT*	1.04	*RPS7*	1.32	*RPS7*	1.14
	7.00	*GAPDH*	1.73	*18S*	1.06	*GAPDH*	1.54	*GAPDH*	1.23
	8.00	*RPS32*	2.41	*RPL32*	2.11	*RPS32*	2.31	*RPS32*	1.53

In different insect tissues, the ΔCt, BestKeeper and geNorm methods identified *TUB* as the most stable reference gene, whereas *RPS13* was optimal using NormFinder (Table 2). With respect to the least stable gene, the four methods were in agreement and indicated that *RPL32* had the lowest expression stability in different tissues (Table 2). According to RefFinder, the stability ranking of the eight reference genes in different insect tissues was *TUB*>*RPS13*>*EF1-α*>*ACT*>*RPS7*>*18S*>*GAPDH*>*RPL32* (Fig 3B).

### Stability of reference genes during abiotic and biotic stress

The ΔCt and NormFinder methods recommended *RPS13* as the most stable reference gene in experiments involving high temperature stress; however, analysis by BestKeeper and geNorm indicated that *RPS7* and *18S* were most stable, respectively (Table 3). All four methods identified *EF1-α* as the least stable reference gene (Table 3). RefFinder analysis indicated the following stability ranking of reference genes for high temperature stress: *RPS13*>*18S*>*RPS7*>*ACT*>*GAPDH*>*TUB*>*RPL32*>*EF1-α* (Fig 3C).

**Table 3:** Stability of reference genes during abiotic and biotic stress.

Conditions	Rank	Δ Ct		BestKeeper		NormFinder		geNorm	
		Gene	stability value	Gene	stability value	Gene	Stability value	Gene	Stability value
High temperature	1.00	*RPS13*	0.47	*RPS7*	0.47	*RPS13*	0.05	*18S*	0.23
	2.00	*18S*	0.47	*RPS13*	0.57	*18S*	0.08	*ACT*	0.23
	3.00	*RPS7*	0.49	*18S*	0.57	*RPS7*	0.10	*RPS13*	0.24
	4.00	*ACT*	0.49	*EF1-α*	0.59	*ACT*	0.14	*RPS7*	0.26
	5.00	*GAPDH*	0.55	*TUB*	0.64	*GAPDH*	0.22	*GAPDH*	0.28
	6.00	*TUB*	0.71	*ACT*	0.71	*TUB*	0.36	*RPL32*	0.36
	7.00	*RPL32*	0.73	*RPL32*	0.81	*RPL32*	0.44	*TUB*	0.45
	8.00	*EF1-α*	1.24	*GAPDH*	0.89	*EF1-α*	0.83	*EF-1α*	0.64
Larval crowding	1.00	*TUB*	0.75	*RPS13*	0.14	*TUB*	0.14	*EF1-α*	0.46
	2.00	*RPS7*	0.76	*18S*	0.36	*RPS7*	0.21	*ACT*	0.46
	3.00	*RPS13*	0.82	*RPS7*	0.37	*RPS13*	0.28	*TUB*	0.53
	4.00	*ACT*	0.89	*TUB*	0.49	*18S*	0.38	*RPS7*	0.63
	5.00	*18S*	0.90	*ACT*	0.66	*ACT*	0.45	*RPS13*	0.68
	6.00	*EF1-α*	0.96	*EF-1α*	0.68	*EF1-α*	0.51	*GAPDH*	0.72
	7.00	*GAPDH*	1.02	*GAPDH*	0.73	*GAPDH*	0.59	*18S*	0.78
	8.00	*RPS32*	1.45	*RPL32*	0.90	*RPL32*	0.96	*RPL32*	0.95
Pesticide exposure	1.00	*EF1-α*	1.33	*GAPDH*	0.30	*EF1-α*	0.22	*RPS7*	0.21
	2.00	*RPS7*	1.36	*RPS7*	0.53	*RPS7*	0.56	*RPL32*	0.21
	3.00	*RPS32*	1.39	*RPL32*	0.56	*RPL32*	0.56	*18S*	0.65
	4.00	*18S*	1.60	*TUB*	1.01	*18S*	0.79	*ACT*	0.80
	5.00	*GAPDH*	1.67	*18S*	1.20	*GAPDH*	0.86	*EF1-α*	0.94
	6.00	*TUB*	1.88	*EF1-α*	1.24	*TUB*	1.03	*GAPDH*	1.05
	7.00	*ACT*	1.93	*RPS13*	1.32	*ACT*	1.11	*TUB*	1.20
	8.00	*RPS13*	1.94	*ACT*	2.08	*RPS13*	1.74	*RPS13*	1.60

In larval crowding trials, ΔCt and NormFinder recommended *TUB* as the most stable reference gene, whereas BestKeeper and geNorm selected *RPS13* and *EF1-α* as most stable, respectively. All four methods identified *RPL32* as the least stable reference gene (Table 3). According to RefFinder, the stability ranking of reference genes for larval crowding was *TUB*>*RPS13*>*RPS7*>*ACT*>*EF1-α>18S>GAPDH>RPL32* (Fig 3D).

In the pesticide exposure experiments, ΔCt and NormFinder indicated that *EF1-α* was the most stable gene, but *GAPDH* and *RPS7* showed optimal stability using BestKeeper and geNorm analysis, respectively. The ΔCt, NormFinder and geNorm methods identified *RPS13* as the least stable gene, whereas BestKeeper identified *ACT* as least stable (Table 3). RefFinder indicated the following stability ranking in pesticide exposure trials: *RPS7*>*EF1-α>RPL32>GAPDH>18S>TUB>RPS13>ACT* (Fig 3E).

### Optimal number of reference genes based on geNorm

**Fig 4:**
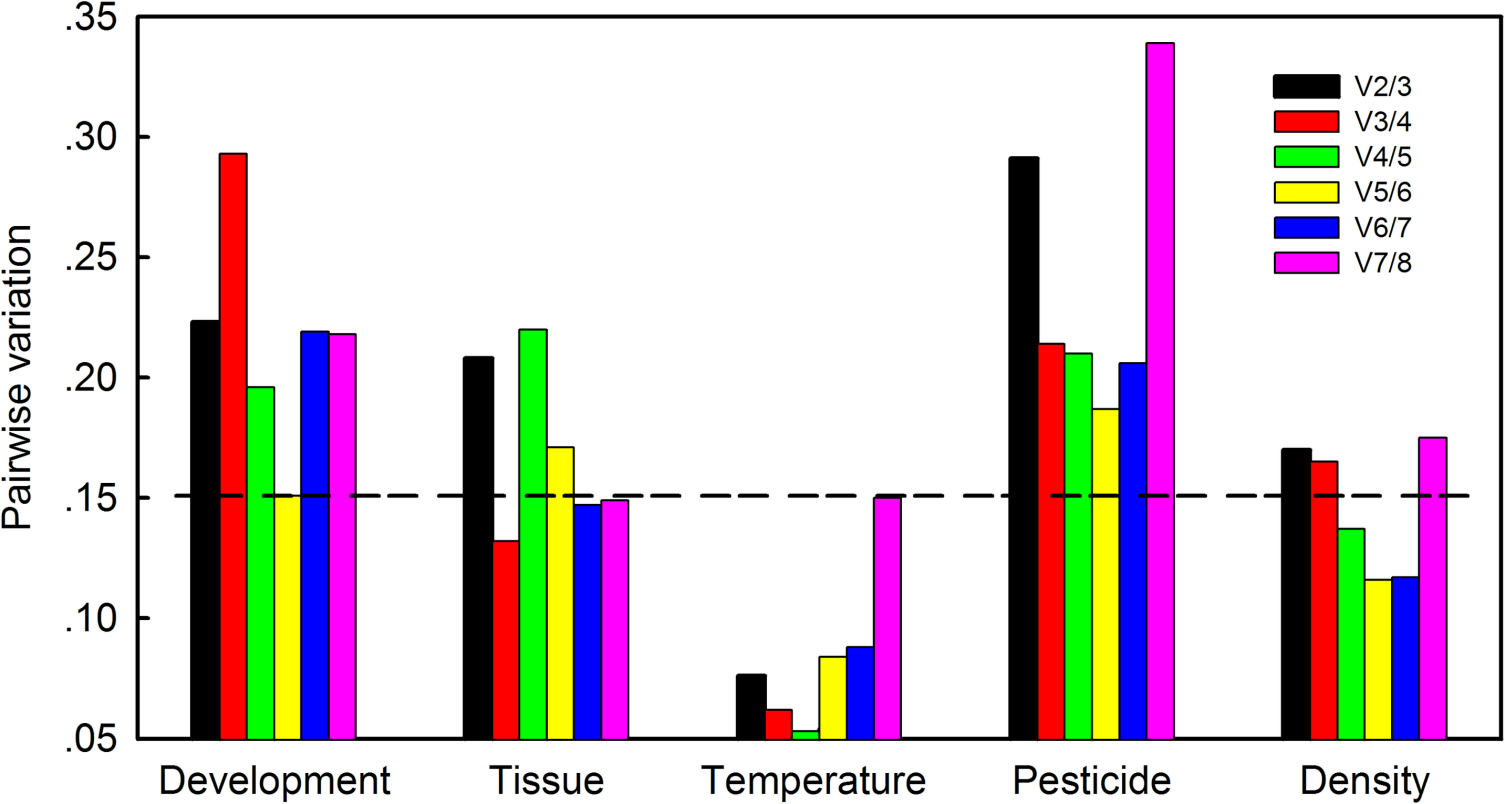
Optimal number of reference genes for normalization of gene expression in *M*. *separata*. Pairwise variation (Vn/Vn+1) was determined between normalization factors (NFn and NFn+1) using the geNorm program. Values less than 0.15 indicated that additional genes were not required for normalization of gene expression.

The geNorm algorithm uses mean expression stability values to determine the optimal number of reference genes for a given experimental condition. geNorm analysis of *M*. *separata* developmental stages and exposure to pesticides resulted in V-values that all exceeded 0.15 (Fig 4), indicating that three reference genes were required for reliable normalization (Table 4). With respect to different tissues of *M*. *separata*, the first value below 0.15 emerged at V3/4, suggesting that three reference genes were required for reliable normalization (Fig 4; Table 4). In high temperature experiments, all stability values were lower than 0.15, thus suggesting that two reference genes were sufficient for normalization (Fig 4; Table 4). In the larval crowding trial, the first value below 0.15 was observed at V4/5, suggesting that four reference genes were required for reliable normalization in larval density experiments (Fig 4; Table 4).

**Table 4:** Recommended reference genes for different experimental conditions.

Experimental conditions	No. reference genes	Recommended reference genes
Developmental stages	3	*ACT*, *EF1-α* and *TUB*
Tissues	3	*TUB*, *RPS13* and *EF1-α*
High temperature	2	*RPS13* and *18S*
Larval crowding	3	*TUB*, *RPS13* and *RPS7*
Pesticide exposure	4	*RPS7*, *EF1-α*, *RPL32* and *GAPDH*

### Validation of selected reference genes using *Mshsp90*

**Fig 5:**
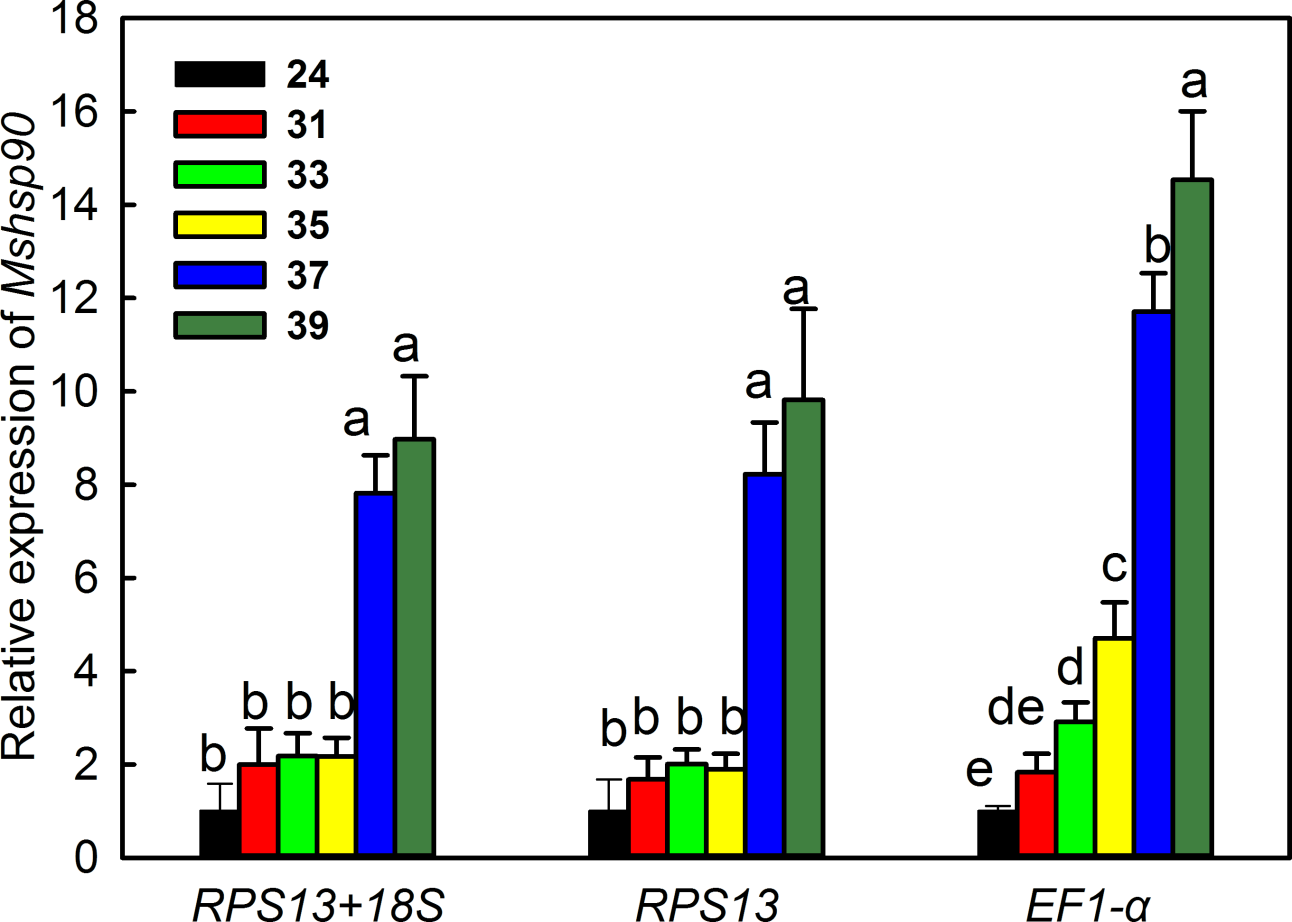
Relative expression of *Mshsp90* during high temperature stress. Relative expression is shown for *Mshsp90* normalized with the most stable reference genes for high temperature stress (*RPS13* and *18S*) and the least stable gene, *EF1-α*. Columns represent mean expression at 24, 31, 33, 35, 37 and 39 ^o^C, vertical bars show the SD of three replications, and columns labeled with different letters represent significant difference at *P ≤* 0.05.

The relative expression of *Mshsp90* was used to validate the recommended reference genes (*RPS13* and *18S*) during exposure of *M*. *separata* to high temperature stress. When *RPS13* and *18S* were used in combination or *RPS13* was used alone to normalize the data, there was no significant difference in *Mshsp90* expression from 24–35°C; however, expression showed a significant, marked increase at 37 or 39°C (*RPS13*+*18S*: *F*_17,5_ = 35.254, *P* = 0.0001; *RPS13*: *F*_17,5_ = 16.112, *P* = 0.0001) (Fig 5). In contrast, when the least stable reference gene, *EF-1α*, was used to normalize the data, *Mshsp90* expression showed a significant increase in expression at 33°C and higher (*F*_17,5_ = 151.511, *P* = 0.0001) (Fig 5). In general, the expression of *Mshsp90* was somewhat lower when the optimal reference genes (*RPS13* and *18S*) were used for normalizing expression as compared to the least stable gene (*EF-1α*) (Fig 5).

## Discussion

qRT-PCR is a widely-used technique for quantifying transcription of target genes in expression studies, which is a critical factor in characterizing gene function [33]. However, the accuracy of target gene expression largely relies on choosing the appropriate internal control, which is commonly known as a reference or ‘housekeeping’ gene. Consequently, it is critical to validate the expression stability of reference gene(s) under different experimental treatments prior to using them to normalize gene expression.

The *M*. *separata* genome has been sequenced, and transcriptomes for insect development, exposure to stressful conditions, and migratory behavior have been described for this species [21–24]. Numerous qRT-PCR studies have been conducted with *M*. *separata* using reference genes utilized for other insect species; however, the use of inappropriate reference genes may lead to misinterpretation of data [34–35]. Therefore, in the current study we used five software programs to evaluate the expression stability of eight candidate reference genes in *M*. *separata* exposed to five different experimental conditions. The mean Ct values of the eight reference genes varied from 13.20 (*18S*) to 24.59 (*GAPDH*), and standard deviations of Ct values ranged from 1.2 (*GAPDH*) to 2.6 (*EF1-α*) (Fig 2). Collectively these results indicated that the reference gene transcripts varied in abundance and the overall expression level could varied considerably among experimental treatments. Thus, it is likely that the expression levels of target genes will also show substantial variation depending on the reference gene and experimental conditions.

With the advent of next-generation sequencing techniques, numerous studies have been conducted to identify and select reference genes in human, animal, plant and insect species [36]. In arthropods, at least 45 species have been investigated for reference gene identification and selection under diverse experimental conditions [37]. These include agricultural and urban pests [2, 7, 37–46], beneficial predators [11, 47], pollinators [48–49], and other economically important insects [2, 50]. These studies concur with our study and provide documentation that reference gene expression varies with the experimental conditions.

Our data demonstrated that four computational methods (e.g. ΔCt, BestKeeper, NormFinder, and geNorm) resulted in different stability rankings for the eight reference genes. For example, ΔCt and geNorm recommended *ACT* as the most stable reference gene for different stages of development, but BestKeeper and NormFinder methods recommended *TUB* and *18S* as the most stable, respectively (Table 2). The ΔCt and NormFinder methods identified *RPS13* as the best reference gene for the high temperatures, while BestKeeper and geNorm identified *RPS7* and *18S* as the most stable genes, respectively (Table 3). These varied results may be attributed to the different algorithms used by the four programs [51]. To address this problem, we used the online software RefFinder [30] to generate the final stability ranking. Analysis using RefFinder indicated that *ACT* was the most stable gene during *M*. *separata* development; *TUB* was the preferred reference gene in different tissues and in the larval crowding trials; and *RPS13* and *RPS7* were the most stable reference genes during exposure to high temperatures and pesticides, respectively (Fig 3).

*ACT*, an important structural protein, is expressed at various levels in lot of cell types. It is considered as the most ideal reference gene in qRT-PCR analysis. Our data indicated that *ACT* was the most stable gene across differenct developmental stages of *M*. *separata*, which is consitent with results found in *Apis mellifera* [52], *Schistocerca gregaria* [37], *Drosophila melanogaster* [53], *Plutella xylostella* and *Chilo suppressalis*[2], *Chortoicetes terminifera*[54], *Diuraphis noxia*[55] *and Liriomyza trifolii* [56]. However, *ACT* has been regarded as an unsuitable reference gene for qRT-PCR analysis of developmental stages of *Frankliniella occidentalis* [43], *Nilaparvata lugens* [57].

*TUB*, a type of cytoskeletal structure protein, is another commonly used reference gene. In this study, *TUB* was considered as the most appropriate reference gene for tissue and larval crowding studies, which is similar to results found in *Locusta migratoria*[58]. To the best of our knowledge, *TUB* has been reported unsuitable to normalize gene expression in the brain of desert locust [37] and in virus-infected planthoppers[59].

Ribosomal protein (RP), a principal component of ribosomes, involves in multiple physiologcial processes of organisms, including intracellular protein biosynthesis, DNA repair, cell differentiation, and so on [60]. Our results suggested that the *RPS13* showed the most stable expression under high temperature conditions, whereas the *RPS7* exhibited the most stable expression under pesticide exposure conditions. These results were similar with privious studies on different developmental stages and photoperiods conditions for *Plutella xylostella*[41] and tissues for feline species[61]. Recently, other memembers of RP gene family have been reported to show the most stable expression in *Tribolium castaneum* (RPS3: [62]), *Spodoptera litura* (*RPS10*: [7]), *N*.*lugens* (*RPS11*: [57]), *Cimex lectularius* (*RPL18*: [63]), *Helicoverpa armigera* (*RPL28*: [64], *F*.*occidentalis* (*RPL32*: [43]) and *Schistocerca gregaria* (*RP49*: [37]).

Additionally, in previous studies, a single reference gene was used to normalize expression of target genes under multiple experimental conditions. In our study, *RPS7* was ranked as fairly stable under high temperature stress, larval crowding, and pesticide exposure (Fig 3C–3E); therefore, *RPS7* could be used as a reference gene for these experimental conditions in *M*. *separata*.

Previous studies have demonstrated that a single reference gene is insufficient to normalize expression data and may lead to inappropriate conclusions regarding the biological function of target genes [42, 65]. Therefore, some researchers have advocated the use of multiple reference genes, and this practice has improved the expression stability of target genes [11, 42, 47]. Our results demonstrated that three reference genes were required for normalizing gene expression in *M*. *separata* developmental stages, tissue types and pesticide exposure trials; two reference genes were sufficient to normalize expression during high temperature stress; and four reference genes were needed to obtain reliable normalization during larval crowding (Fig 4).

To further validate the reference genes in *M*. *separata*, the expression of *Mshsp90* was evaluated during high temperature stress. *hsp90* functions as a molecular chaperone and plays multiple roles in the stress response of many organisms. Our results suggested that relative expression level of *Mshsp90* was relatively constant from 24 to 35°C, but significantly up-regulated at 37 and 39°C when normalized by the recommended reference gene, *RPS13* (Fig 5). A similar result was also observed when expression data were normalized using a combination of reference genes, e.g. *RPS13* and *18S* (Fig 5). However, when *Mshsp90* expression data was normalized with the least stable reference gene, *EF1-α*, expression showed a significant upregulation at lower temperatures (33 and 35°C) (Fig 5). These results showed that the arbitrary selection of a reference gene may lead to misleading results regarding the function of a target gene. Therefore, the choice of optimal reference genes is essential for determining the accuracy of expression results, especially for subtle differences. To improve the accuracy of results, it may be necessary to use a panel of selected reference genes for each experimental condition.

## Conclusions

In summary, we used five software programs to evaluate the expression stability of eight reference genes under different biotic and abiotic conditions. Based on our comprehensive analysis, different reference genes and combinations of reference genes should be used to normalize gene expression in *M*. *separata* subjected to different experimental conditions. Our results provide reliable normalization of qRT-PCR data and also lay a foundation for future functional studies of target gene expression in *M*. *separata*.
